# Phosphorylation of the plant photoreceptor UVR8 regulates its dimer/monomer status and enhances UV-B signaling across species

**DOI:** 10.1093/plcell/koag285

**Published:** 2026-10-08

**Authors:** Boqin Tong, MeLongying Wangzha, Jie Wang, Baoguo Wu, Chunli Zhang, András Viczián, Assaf Mosquna, Yizhuo Mu, Bingbing Wan, Li Lin, Ruohe Yin

**Affiliations:** School of Agriculture and Biology, Shanghai Jiao Tong University, 800 Dongchuan Road, Minhang District, Shanghai 200240, China; School of Agriculture and Biology, Shanghai Jiao Tong University, 800 Dongchuan Road, Minhang District, Shanghai 200240, China; School of Agriculture and Biology, Shanghai Jiao Tong University, 800 Dongchuan Road, Minhang District, Shanghai 200240, China; School of Agriculture and Biology, Shanghai Jiao Tong University, 800 Dongchuan Road, Minhang District, Shanghai 200240, China; School of Agriculture and Biology, Shanghai Jiao Tong University, 800 Dongchuan Road, Minhang District, Shanghai 200240, China; Laboratory of Photo and Chronobiology, Institute of Plant Biology, Biological Research Centre, Hungarian Research Network (HUN-REN), Temesvári krt. 62, Szeged H-6726, Hungary; The Robert H. Smith Institute of Plant Sciences and Genetics in Agriculture, The Hebrew University of Jerusalem, Herzl 229, Rehovot 7610000, Israel; Section of Horticulture, School of Integrative Plant Science, Cornell University, 220 Bruckner Hall, Ithaca, NY 14853, United States; Key Laboratory of Systems Biomedicine (Ministry of Education), Shanghai Center for Systems Biomedicine, 800 Dongchuan Road, Minhang District, Shanghai Jiao Tong University, Shanghai 200240, China; School of Agriculture and Biology, Shanghai Jiao Tong University, 800 Dongchuan Road, Minhang District, Shanghai 200240, China; School of Agriculture and Biology, Shanghai Jiao Tong University, 800 Dongchuan Road, Minhang District, Shanghai 200240, China; Key Laboratory of Urban Agriculture Ministry of Agriculture, 800 Dongchuan Road, Minhang District, Shanghai Jiao Tong University, Shanghai 200240, China

## Abstract

Ultraviolet-B (UV-B) is an integral component of sunlight that profoundly shapes plant growth, development, and survival. Plant photoreceptor UV RESISTANCE LOCUS 8 (UVR8) homodimers perceive UV-B and dissociate into active monomers to initiate signaling, yet how UVR8 dimer stability and monomer activity are post-translationally regulated remains unclear. We identify UV-B-enhanced phosphorylation at threonine 356 of UVR8, which promotes UV-B-induced UVR8 dimer dissociation and delays redimerization, shifting dimer/monomer equilibrium towards active monomer in Arabidopsis. Importantly, the same regulatory mechanism is conserved in tomato, demonstrating that phosphorylation at this site represents a broadly applicable molecular switch across species. Moreover, this modification enhances the binding of UVR8 to CONSTITUTIVE PHOTOMORPHOGENIC 1 (COP1), prolongs UVR8 nuclear residence, and amplifies UV-B signaling outputs. Together, these findings uncover phosphorylation at T356 as an evolutionarily conserved molecular switch that fine-tunes photoreceptor conformational change and signaling, highlighting phosphorylation as a critical posttranslational control of light perception in plants.

## Teaser

Phosphorylation at a threonine residue of UVR8 facilitates UV-B-induced UVR8 dimer dissociation and inhibits monomer redimerization shifting dimer/monomer equilibrium towards monomer, which is important to amplify UV-B signaling.

## Introduction

Sunlight is a critical environmental factor that shapes plant growth and development, with ultraviolet-B (UV-B; 280–315 nm) radiation comprising a small but biologically highly active fraction of the solar spectrum reaching the Earth's surface. On the one hand, UV-B serves as an important environmental cue that regulates developmental programs, photomorphogenesis, and stress acclimation responses (Jenkins 2014; Fernández et al. 2016; Liang et al. 2019; Podolec et al. 2021a; Chen et al. 2022). On the other hand, excessive UV-B exposure can generate DNA lesions, induce protein denaturation, and disrupt photosynthetic efficiency, thereby compromising plant growth and survival (Hideg et al. 2013; Shi and Liu 2021). To sense and respond to distinct wavelengths of light, plants rely on specialized photoreceptors (Galvão and Fankhauser 2015; Wang and Lin 2020). Among these, UV RESISTANCE LOCUS 8 (UVR8) is the sole identified UV-B photoreceptor, functioning as the central regulator of UV-B-induced signaling and stress acclimation (Kliebenstein et al. 2002; Brown et al. 2005; Rizzini et al. 2011; Hideg et al. 2013; Fernández et al. 2016; Liu et al. 2025). UVR8 is involved in responses to other stimuli, including osmotic stress (Fasano et al. 2014; Jansen et al. 2022) and UV-A light (Rai et al. 2020), and inhibits thermo-morphogenesis (Hayes et al. 2017), shade-avoidance responses (Hayes et al. 2014), and promotes herbicide resistance (Groves and Franklin 2025).

In its ground state, UVR8 exists as a homodimer, which dissociates into active monomers upon UV-B exposure to initiate signaling (Rizzini et al. 2011). Structural analyses revealed that dimer stability is maintained by salt bridges between Arg and Asp residues and by charge-stabilized hydrogen bonds at the dimer interface (Christie et al. 2012; Wu et al. 2012). UVR8 uses tryptophan residues as intrinsic chromophores for UV-B perception. Upon UV-B absorption by tryptophan, particularly those positioned at the dimer interface, leads to the destabilization of salt bridges, thereby triggering monomerization and UVR8 activation (Christie et al. 2012; Wu et al. 2012; Li et al. 2022). UVR8 monomers can bind to each other to form dimers in the absence of UV-B (Christie et al. 2012; Wu et al. 2012; Heijde and Ulm 2013; Heilmann and Jenkins 2013). The negative regulators REPRESSOR OF UV-B PHOTOMORPHOGENESIS 1 (RUP1) and RUP2, particularly RUP2, interact with UVR8 and promote redimerization to inhibit UV-B signaling (Heijde and Ulm 2013; Wang et al. 2023). The photocycle of UVR8, namely monomerization and redimerization, is critical for UVR8 activity. In the natural environment, UVR8 does not simply switch from dimer to monomer following UV-B treatment but forms a dimer/monomer equilibrium that is dependent on the relative rates of monomerization and reversion to the dimer (Findlay and Jenkins 2016; Moriconi et al. 2018). Mutational studies further highlight key residues for maintaining UVR8 structure stability: substitution of Trp-285 with phenylalanine generates a stable dimer that fails to monomerize in response to UV-B, whereas replacement with alanine constitutively activates UV-B signaling (Rizzini et al. 2011; Christie et al. 2012; Wu et al. 2012). Similarly, mutation of Gly-101 reduces UVR8 dimer stability, and mutation of Asp-96/Asp-107 leads to dissociation of the UVR8 dimer into monomers in the absence of UV-B (Heilmann et al. 2016; Podolec et al. 2021b). Together, these findings underscore that certain residues play critical roles in affecting UVR8 conformation and signaling activity. Currently, whether post-translational modification of key residues affecting UVR8 dimer stability and signaling remains unknown.

UVR8 protein is located in the cytosol, and UV-B exposure rapidly drives its nuclear translocation and protein interactions that activate downstream signaling (Kaiserli and Jenkins 2007; Liu and Jenkins, 2025). A central component of this pathway is the E3 ligase CONSTITUTIVELY PHOTOMORPHOGENIC 1 (COP1) (Oravecz et al. 2006). Monomerized UVR8 interacts with and inactivates COP1, releasing COP1 substrates such as ELONGATED HYPOCOTYL 5 (HY5), which in turn orchestrates the expression a set of UV-B target genes, leading to inhibition of hypocotyl elongation, anthocyanin accumulation and UV-B stress acclimation (Brown and Jenkins 2008; Favory et al. 2009; Cloix et al. 2012; Yin et al. 2015; Lau et al. 2019). Moreover, COP1 binding is required for UV-B-induced UVR8 nuclear accumulation and for its nuclear retention post UV-B irradiation (Qian et al. 2016; Yin et al. 2016; Fang et al. 2022; Zhang et al. 2023a). RUP1/2 compete with COP1 for UVR8 binding, thereby inhibiting UVR8 nuclear retention and promoting its nuclear export (Fang et al. 2022). Mutation at a key residue of COP1, namely C509, responsible for binding to UVR8, attenuates UVR8 binding and UVR8 nuclear accumulation (Zhang et al. 2023a). The interaction with COP1 is critical for UVR8 nucleocytoplasmic distribution and UV-B signaling, however, whether protein post-translational modification regulates COP1 binding and UVR8 nuclear localization remains unclear.

Protein phosphorylation represents a ubiquitous post-translational modification that regulates protein structure, activity, stability, interaction, and localization across cellular compartments (Zhang et al. 2023b). Recent work has revealed that Arabidopsis UVR8 is phosphorylated in vivo: phosphorylation at serine 402 enhances UV-B-induced flavonoid biosynthesis, although UV-B core signaling, such as UVR8 dimer/monomer conformational change and *HY5* gene expression, remains unaffected (Liu et al. 2024). Here, we demonstrate that UVR8 phosphorylation occurs in both the cytosol and nucleus, with nuclear UVR8 exclusively existing in phosphorylated form. UV-B strongly promotes phosphorylated UVR8 nuclear accumulation. Through mass spectrometry and functional analyses, we identify an evolutionarily well-conserved phosphorylation site, threonine 356 (T356), located at the surface of the UVR8 dimer structure. UV-B-enhanced phosphorylation at T356 facilitates UVR8 dimer dissociation and delays monomer redimerization, shifts photo-equilibrium towards monomer, enhances UVR8-COP1 interaction, and promotes UVR8 nuclear localization. These findings establish T356 phosphorylation as a critical regulatory mechanism that coordinates UVR8 dimer/monomer equilibrium, protein interactions, subcellular dynamics, and signaling amplification.

## Results

### UV-B promotes nuclear accumulation of phosphorylated UVR8

UVR8 phosphorylation has been detected by ^32^P labeling in Arabidopsis, but standard SDS-PAGE failed to resolve modified forms, suggesting that phosphorylation affects only a minor pool of the protein (Liu et al. 2024). Using phos-tag SDS-PAGE, we resolved 2 UVR8 species in wild-type seedlings, of which the slower-migrating band was eliminated by λ-phosphatase treatment, confirming it as phosphorylated UVR8 (Fig. 1a). UV-B irradiation increased the abundance of this phosphorylated species, raising its proportion from ∼10.5% under white light to ∼22.9% under UV-B (Fig. 1a, b).

**Figure 1:**
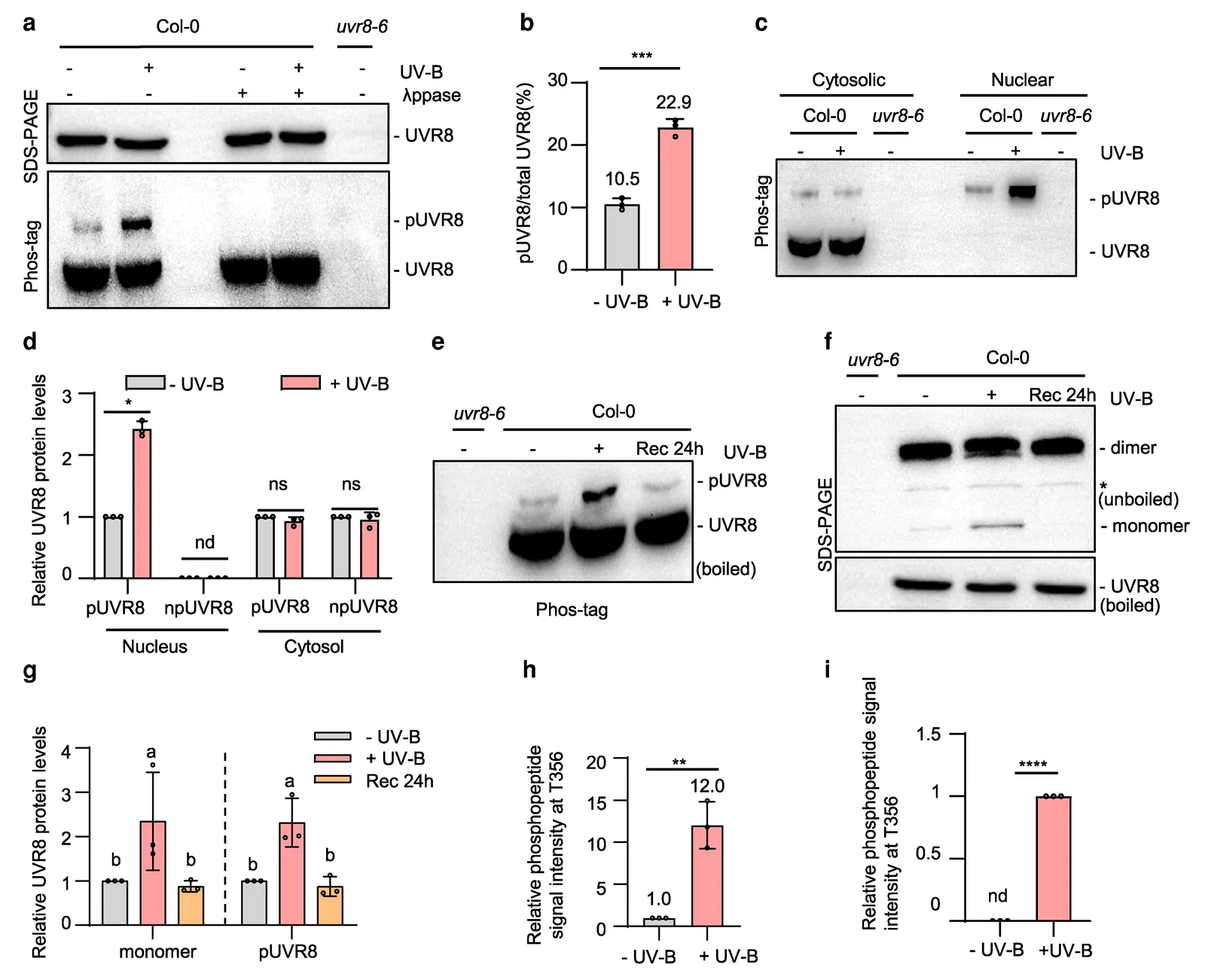
UV-B promotes nuclear accumulation of phosphorylated UVR8. (a, b) UV-B enhances the phosphorylation of total UVR8. (A) Immunoblot analyses for UVR8 levels in *uvr8-6* and wild-type (Col-0) plants grown in white light without (−UV-B) or with UV-B (+UV-B) for 4 d and total protein extracts treated without (−) or with (+) λppase and then analyzed via SDS–PAGE (*upper panel*) and Phos-tag PAGE (*lower panel*). (b) The percentage of phosphorylated UVR8 in plants grown in white light without (−UV-B) or with UV-B (+UV-B) for 4 d. Relative band intensity of phosphorylated and nonphosphorylated UVR8 from Phos-tag immunoblots shown in (a) and other two experiments are quantified. Mean and SD from 3 experiments are shown. (c, d) UV-B promotes nuclear accumulation of phosphorylated UVR8. (c) Immunoblot analyses for UVR8 in cytosolic fraction and nuclear fraction of 4-d-old wild-type (Col-0) and *uvr8-6* plants grown in white light without (−) or with UV-B(+). This experiment has been repeated at least 3 times with similar results. (d) Relative quantification of phosphorylated UVR8 proteins (pUVR8) and nonphosphorylated UVR8 proteins (npUVR8) levels in nucleus and cytosol. pUVR8 and npUVR8 in nucleus and cytosol under UV-B were normalized against corresponding form of UVR8 under white light in the respective compartment. nd, nondetectable. Mean and SD from 3 experiments are shown. (e, g) UV-B affects UVR8 phosphorylation dynamic. Wild-type (Col-0) seedlings were grown either in white light (−) or white light supplemented with UV-B (+), or UV-B-treated plants were recovered under white light for 24 h. UVR8 phosphorylation status was detected in phos-tag PAGE (e), and UVR8 protein levels and dimer/monomer status were detected in SDS–PAGE (f), with an anti-UVR8 antibody. The asterisk in f indicates a cross-reacting band. (g) Relative quantification of phosphorylated and monomeric UVR8 levels as shown in (e) and (f), respectively. Relative quantification was performed by comparing UVR8 levels to that in minus UV-B. Mean and SD from 3 experiments are shown. (h) Total protein mass spectrometry analyses for relative phosphopeptide signal intensity at T356 residue of UVR8 in wild-type plants grown in white light without (−UV-B) or with UV-B (+UV-B) for 4 d. The relative intensities were normalized against that in −UV-B conditions. (i) Nuclear fraction protein mass spectrometry analyses for relative phosphopeptide signal intensity at T356 of UVR8. Seedlings were grown as in (h). The intensities in −UV-B conditions were nondetected (nd), in +UV-B conditions was set to 1. Mean and SD from 3 biological replicates are shown. Statistical significance in (b), (d), (h), and (i) was determined by Student’s *t*-test (*P < 0.05; **P < 0.01; ***P < 0.001; ****P < 0.0001; ns, not significant), in (g) was performed by 1-way ANOVA with different letters indicating significant differences (*P* < 0.05).

Fractionation experiments showed that UV-B elevated nuclear UVR8 levels, consistent with previous reports (Figure S1) (Kaiserli and Jenkins 2007; Qian et al. 2016; Yin et al. 2016; Fang et al. 2022; Zhang et al. 2024). Strikingly, phos-tag immunoblots revealed that cytosolic UVR8 was mostly nonphosphorylated, whereas nuclear UVR8 was exclusively phosphorylated (Fig. 1c). UV-B further boosted nuclear phosphorylated UVR8 levels by ∼2.5-fold compared with white light (Fig. 1c, d). Thus, UV-B selectively promotes the accumulation of phosphorylated UVR8 in the nucleus.

Next, we asked which conformer of UVR8 is phosphorylated in plants. Non-denatured protein samples could not be properly analyzed on a phos-tag gel with our immunoblot system, as it generates very strong background (Figure S2a). Since nuclear UVR8 under UV-B exists mainly, if not exclusively, as the monomeric form, as shown previously (Fang et al. 2022), and UV-B enhances phosphorylated UVR8 accumulation in the nucleus (Fig. 1c), suggesting that the monomer in the nucleus is phosphorylated. To test whether UVR8 homodimer can be phosphorylated in plants, we analyzed UVR8 phosphorylation with previously established constitutively dimeric (UVR8^W285F^) and monomeric (UVR8^W285A^) UVR8 variants. Notably, both mutants exhibited phosphorylation levels comparable to those of wild-type under white light, and their phosphorylation status was not further elevated by UV-B treatment, most likely because these mutants are nonresponsive to UV-B (Figure S2b, c). Importantly, these results demonstrate that both monomeric and dimeric forms of UVR8 can be phosphorylated, and that the phosphorylation of T356 does not strictly depend on the monomerization state, although UV-B enhances overall UVR8 phosphorylation. Together, these results demonstrate that UV-B enhances phosphorylation of a small UVR8 subpopulation and drives its nuclear accumulation, establishing phosphorylated monomer as the active form of UVR8 in signaling.

To examine the reversibility of UVR8 phosphorylation, we included a 24-hour recovery period after UV-B treatment. Both UVR8 phosphorylation levels and monomer abundance returned to baseline after recovery (Fig. 1e–g), indicating that UVR8 phosphorylation is dynamically regulated and correlates with the UVR8 monomeric status, ie, phosphorylation increases upon UV-B-induced monomerization and decreases upon redimerization during recovery.

### UV-B-enhanced phosphorylation of UVR8 threonine 356 in Arabidopsis

To map UVR8 phosphorylation sites, we enriched UVR8 from seedlings grown under white light or white light plus UV-B by immunoprecipitation with a UVR8 antibody and analyzed the products by mass spectrometry. Across 3 independent experiments, we identified 10 phosphorylated residues with a high confidence signal-to-noise ratio (SNR) greater than 5 (false discovery rate ≤1%; Figure S3a). Eight residues were detected both under white light and UV-B conditions, with partial overlap. Among these, threonine 356 (T356) was consistently detected across all the 3 experiments (Figure S3b). Quantification of the relative phosphopeptide signal intensity at the T356 residue from total protein extracts by mass spectrometry indicated that phosphorylation of T356 was strongly enhanced by UV-B to about 12-fold higher than that under white light (Fig. 1h, Figure S4a, b). Furthermore, mass spectrometry analysis of nuclear proteins from Arabidopsis revealed that T356 phosphorylation was essentially undetectable under white light, but was clearly induced upon UV-B treatment in the nucleus (Fig. 1i, Figure S4c). Sequence alignment revealed that T356 is highly conserved in UVR8 orthologs ranging from unicellular algae, across land plants, and even to the human UVR8-related RCC1 protein (Figure S5). These results identify T356 as a major UV-B light-induced and evolutionarily conserved phosphorylation site, which we focused on in subsequent analyses.

### Phosphorylation of threonine 356 enhances UVR8 activity

To test the function of T356 phosphorylation, we generated *uvr8-6* transgenic lines expressing YFP-UVR8, the phosphomimic YFP-UVR8^T356D^ (YFP-T356D), or the phospho-null YFP-UVR8^T356A^ (YFP-T356A) at comparable protein levels (Fig. 2a, b). UV-B induced higher expression of *HY5* and *CHS* (encoding Chalcone Synthase) in the YFP-T356D lines compared with YFP-UVR8 and YFP-T356A lines, and this effect was mirrored by higher CHS protein accumulation and enhanced anthocyanin biosynthesis (Fig. 2c, d; Figure S6a–c). Morphologically, UV-B caused stronger hypocotyl elongation inhibition in YFP-T356D seedlings than in YFP-UVR8 or YFP-T356A lines (Fig. 2e, f). We performed long-term growth experiments in which the transgenic lines were grown under white light with or without UV-B supplementation for 25 d (Figure S7). Notably, YFP-T356D plants exhibited a significantly reduced rosette diameter compared to both YFP-UVR8 and YFP-T356A plants, indicating that the UV-B hypersensitive phenotype of YFP-T356D extends to long-term growth performance (Figure S7a, b). Finally, YFP-T356D seedlings exhibited improved UV-B stress tolerance following acclimation, whereas all lines responded similarly without acclimation (Fig. 2g; Figure S8). Together, these results demonstrate that phosphorylation of T356 potentiates UVR8 signaling outputs, enhancing transcriptional activation, photomorphogenic responses, and UV-B stress tolerance.

**Figure 2:**
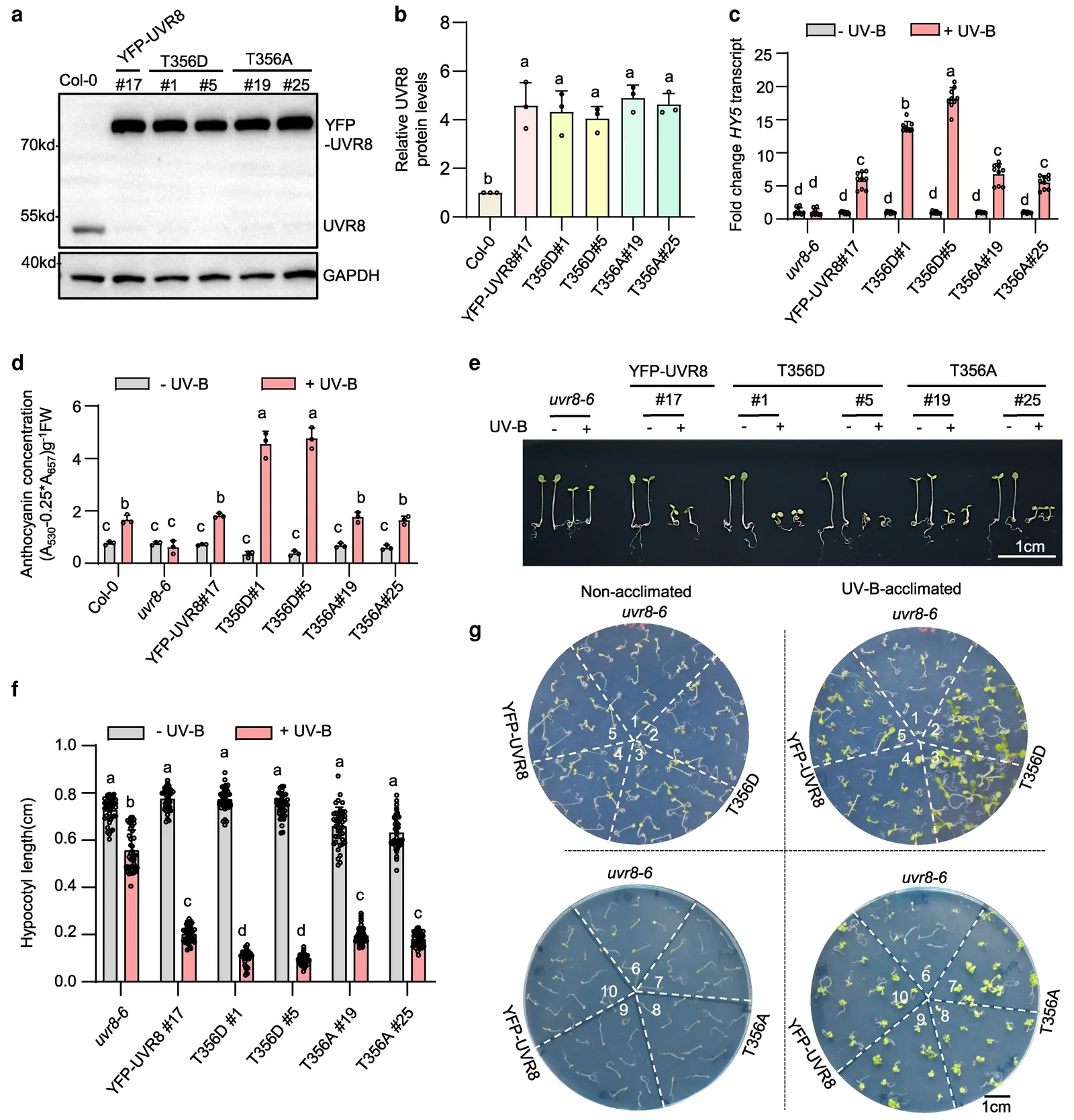
Phosphorylation of UVR8 T356 promotes UV-B signaling and UV-B stress tolerance. (a, b) UVR8 fusion protein levels are similar in 4-d-old YFP-UVR8 (*35S:YFP-UVR8/uvr8-6*), T356D (*35S:YFP-UVR8^T356D^/uvr8-6*), and T356A (*35S:YFP-UVR8^T356A^/uvr8-6*) transgenic lines. (a) Immunoblot analyses for UVR8 in 4-d-old transgenic lines grown under white light with anti-UVR8^426-440^ and anti-GAPDH (loading control) antibodies. (b) Relative UVR8 protein levels were determined against that of Col-0. Mean and SD from 3 experiments are shown. (c) RT-qPCR analysis of *HY5* mRNA of 4-d-old seedlings irradiated for 1 h with (+) or without (−) UV-B, relative to the respective line without UV-B; means with SD, *n* = 3. (d) Quantification of anthocyanin levels of 4-d-old seedlings grown under white light (−UV-B) or white light supplemented with UV-B (+UV-B). Means with SD, *n* = 3. (e, f) Hypocotyl growth in white light with or without UV-B. Images of representative individuals (e) and hypocotyl lengths (f) of 4-d-old seedlings; means with SD; *n* = 40. Scale, 1 cm. (g) UV-B stress tolerance of non-UV-B-acclimated and UV-B-acclimated (pretreated with narrowband UV-B for 24 h) 4-d-old seedlings treated for 3 h with broadband UV-B. After UV-B stress treatment, the seedlings were allowed to recover for 7 d under white light before the image was captured. Representative images of 3 consistent experiments are shown. Genotypes in each plate: *uvr8-6* (1,6), YFP-T356D line #1 (2) and #5 (3), YFP-T356A line #19 (7) and #25 (8), and YFP-UVR8 line #17(4,5,9,10). Scale, 1 cm. Statistical analyses in (b), (c), (d), and (f) were performed by 2-way ANOVA with different lowercase letters indicating significant differences (*P* < 0.05).

### T356 phosphorylation facilitates UV-B-induced UVR8 dimer dissociation and shifts UVR8 dimer/monomer equilibrium to monomer

We next examined whether T356 phosphorylation affects the UVR8 dimer-to-monomer switch, the key step in receptor activation. In transgenic Arabidopsis, both YFP-UVR8 and YFP-T356A formed homodimers under white light and monomerized in response to UV-B, whereas YFP-T356D was detected as a monomer irrespective of light conditions on SDS-PAGE denaturing gels (Fig. 3a). To confirm the effect of T356 phosphorylation, T356 was substituted with glutamic acid (E), which is another way to mimic phosphorylation. Similar results were obtained with untagged UVR8^T356E^ lines, confirming that phosphorylation at T356 facilitates dimer dissociation (Fig. 3b). Native gel immunoblots and size-exclusion chromatography indicated that both phosphomimic and wild-type UVR8 proteins can form dimers and retain UV-B-induced monomerization capacity, suggesting that phosphorylation reduces dimer stability rather than abolishing dimer formation (Fig. 3c–e). Moreover, we noted that UVR8^T356D^ and UVR8 show largely overlapping SEC elution profiles, both predominantly dimeric in the absence of UV-B. Upon UV-B treatment, however, UVR8^T356D^ retained a lower dimeric shoulder than UVR8, suggesting its stronger monomerization than UVR8 under equivalent UV-B conditions. Notably, following UV-B treatment, the amplitude of the monomer peak appeared higher relative to the dimer peak in T356D, but the amplitudes for both monomer and dimer peaks were similar in the wild-type protein. Next, we compared monomerization kinetics between YFP-UVR8 and YFP-T356D in vivo. Following prolonged UV-B treatments, both proteins exhibited enhanced monomerization, but YFP-T356D consistently appeared exclusively as monomers, whereas YFP-UVR8 retained residual dimers (Fig. 3f, g). Together, these results demonstrate that phosphorylation at T356 facilitates UV-B-induced UVR8 dimer dissociation and shifts the dimer/monomer photo-equilibrium to monomer.

**Figure 3:**
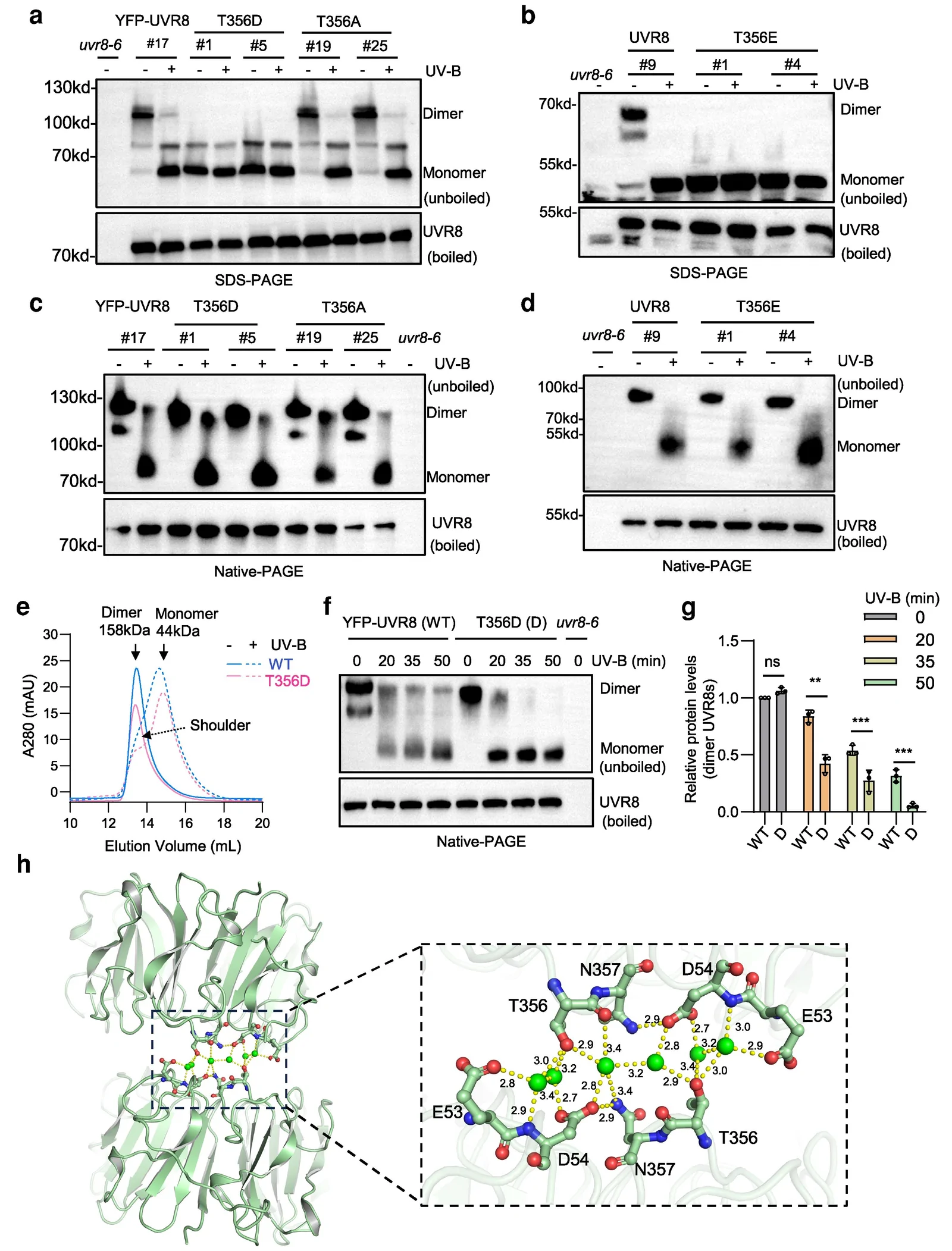
Phosphorylation of T356 promotes UVR8 dimer dissociation. (a, b) Phosphomimic T356 by either Aspartate (T356D) or Glutamate (T356E) dissociate UVR8 dimer into monomer under white light on denaturing SDS–PAGE. Four-day-old *uvr8-6, 35S:YFP-UVR8/uvr8-6* (#17), *35S:YFP-UVR8^T356D^/uvr8-6* (#1, #5), *35S:YFP-UVR8^T356A^/uvr8-6* (#19, #25), *35S: UVR8/uvr8-6 (#9) and 35S: UVR8^T356E^/uvr8-6* (#1, #4) grown under white light and irradiated without (−) or with broadband UV-B for 30 min (+). Dimer for wild-type UVR8 (YFP-UVR8 and also UVR8) and phosphonull UVR8 (YFP-UVR8^T356A^) were detectable in nonheat-denatured samples. Parallel denatured samples indicate equal amounts of UVR8 protein. Immunoblot analyses were performed with anti-GFP (a) and anti-UVR8^410-424^ antibodies (b), respectively. (c, d) Phosphomimic T356 by either Aspartate (c) or Glutamate (d) maintain normal UVR8 dimer under white light and dissociate into monomer under UV-B on native PAGE. Experimental conditions are identical to that of (a) and (b) except that native PAGE are used in (c) and (d). (e) Size-exclusion chromatography of His-UVR8 and His-UVR8^T356D^ proteins purified from *E. coli*, and irradiated without (−) and with broadband UV-B for 60 min (+). (f, g) YFP-UVR8^T356D^ is more sensitive to UV-B than YFP-UVR8. (f) YFP-T356D has faster UV-B-induced dimer dissociation than YFP-UVR8. Four-day-old YFP-UVR8 (#1), YFP-UVR8^T356D^ (#5), and *uvr8-6* plants were grown in white light and irradiated with broadband UV-B for the indicated time to induce UVR8 dimers dissociate into monomers. Dimers for both wild-type and phosphomimic UVR8 (YFP-UVR8^T356D^) were detectable in nonheat-denatured samples on native PAGE. Parallel denatured samples indicate equal amounts of UVR8 protein. Immunoblot analyses were performed with anti-UVR8^410-424^ antibody. (g) Relative UVR8 dimer protein levels were determined against that of YFP-UVR8 dimer without UV-B (0 min). Mean and SD are shown (*n* = 3). Statistical analyses were performed by Student’s *t*-test (ns, no significance; **P < 0.01; ***P < 0.001). (h) UVR8 dimer showing water-mediated hydrogen band network between T356 and 3 amino acids (D54, E53, and N357) at the dimeric interface. The schematic depicts the interface of UVR8 dimer, colored in light green, and the inset shows detailed water mediated comprehensive hydrogen bonds network. Water molecules are colored in dark green and hydrogen bonds are shown as dash lines. The distances between atoms are labeled in Å (10^−10^ m).

UVR8 has been characterized in several crops (Fernández et al. 2020; Liu et al. 2020; Chen et al. 2024; Hu et al. 2024; Wu et al. 2026). We further tested whether this mechanism is conserved in tomato (*Solanum Lycopersicum*), which is a widely used model plant in photomorphogenesis research. Transgenic lines expressing SlUVR8-GFP or the phosphomimic SlUVR8^T360D^-YFP were generated in the *Sluvr8* knock-out mutant background (Liu et al. 2020). Two independent SlUVR8^T360D^-YFP tomato lines were selected (Figure S9a, b). SlUVR8-YFP forms homodimers under white light and monomerizes under UV-B on SDS-PAGE. In contrast, both dimeric and monomeric SlUVR8^T360D^-YFP existed under white light, and SlUVR8^T360D^-YFP dimer readily monomerized under UV-B (Figure S9c, d). Thus, phosphorylation of SlUVR8^T360^ also promotes SlUVR8 dimer dissociation in tomato (Figure S9c, d). SlUVR8^T360D^-YFP seedlings displayed stronger UV-B-induced hypocotyl inhibition, anthocyanin biosynthesis, and induction of *SlHY5* and *SlCHS1* than SlUVR8-YFP (Figure S9e–i), indicating enhanced UV-B signaling. These findings indicate that phosphorylation at T356/T360 is a conserved regulatory mechanism across different plant species.

According to the resolved UVR8 structure (Christie et al. 2012; Wu et al. 2012), T356 is located at the surface of the dimer interface within a loop region (Fig. 3h). Interestingly, T356 establishes a comprehensive hydrogen-bond network, which is mediated by several water molecules in the dimer interface with adjacent D54, E53, and N357 residues. Among all identified phosphorylation sites from the UVR8 core fragment (not including those in the flexible N- and C-termini), only T356 and S105 reside at the dimer interface (Figure S10a). To assess whether S105 phosphorylation also influences UVR8 dimer/monomer status, we generated Arabidopsis plants expressing YFP-UVR8^S105A^ or YFP-UVR8^S105D^ in the *uvr8-6* mutant background. Both variants remained dimeric on SDS-PAGE under non-UV-B conditions (Figure S10b), indicating that phosphorylation of S105, not like that of T356, does not strongly impair UVR8 dimer/monomer status. Previous studies showed that Arg residues at the dimer interface contribute to dimer formation through salt bridges, such as those formed between R354 and E43 or R354 and E53 (Christie et al. 2012; Wu et al. 2012). Structural analyses further place T356 in close proximity to R354 (Figure S10c, d). Phosphorylation of T356 introduces negative charges near R354, likely perturbing the loop conformation, weakening salt bridge interactions, and widening the monomer-monomer interface, thereby facilitating the UV-B-induced UVR8 dimer dissociation and likely also affecting UVR8 binding to other proteins. To test this hypothesis, we performed split-luciferase assays comparing COP1 interaction with the T356D single mutant versus the T356D/R354A double mutant under both –UV-B and +UV-B conditions. The results showed that the T356D/R354A double mutant exhibited significantly stronger COP1 interaction than the T356D single mutant, and this trend was consistent under both light conditions (Figure S11). Notably, the T356D/R354A double mutant interacted with COP1 already under white light. This supports the notion that the conformational perturbation has functional consequences extending beyond UVR8 dimerization.

### Phosphorylation of T356 enhances UVR8-COP1 interaction

UV-B-activated UVR8 interacts with COP1 to initiate signaling (Cloix et al. 2012). Yeast two-hybrid assays showed that UVR8^T356D^ exhibited a stronger UV-B-induced interaction with COP1 than wild-type UVR8, whereas UVR8^T356A^ showed a slight reduction (Fig. 4a). Split-luciferase assays in *Nicotiana Benthamiana* leaves confirmed these results, with UVR8^T356D^ consistently displaying stronger COP1 binding (Figure S12). Co-immunoprecipitation in transgenic Arabidopsis lines further demonstrated that YFP-UVR8^T356D^ interacts more strongly with COP1 than YFP-UVR8 or YFP-UVR8^T356A^ under UV-B (Fig. 4b, c; Figure S13).

**Figure 4:**
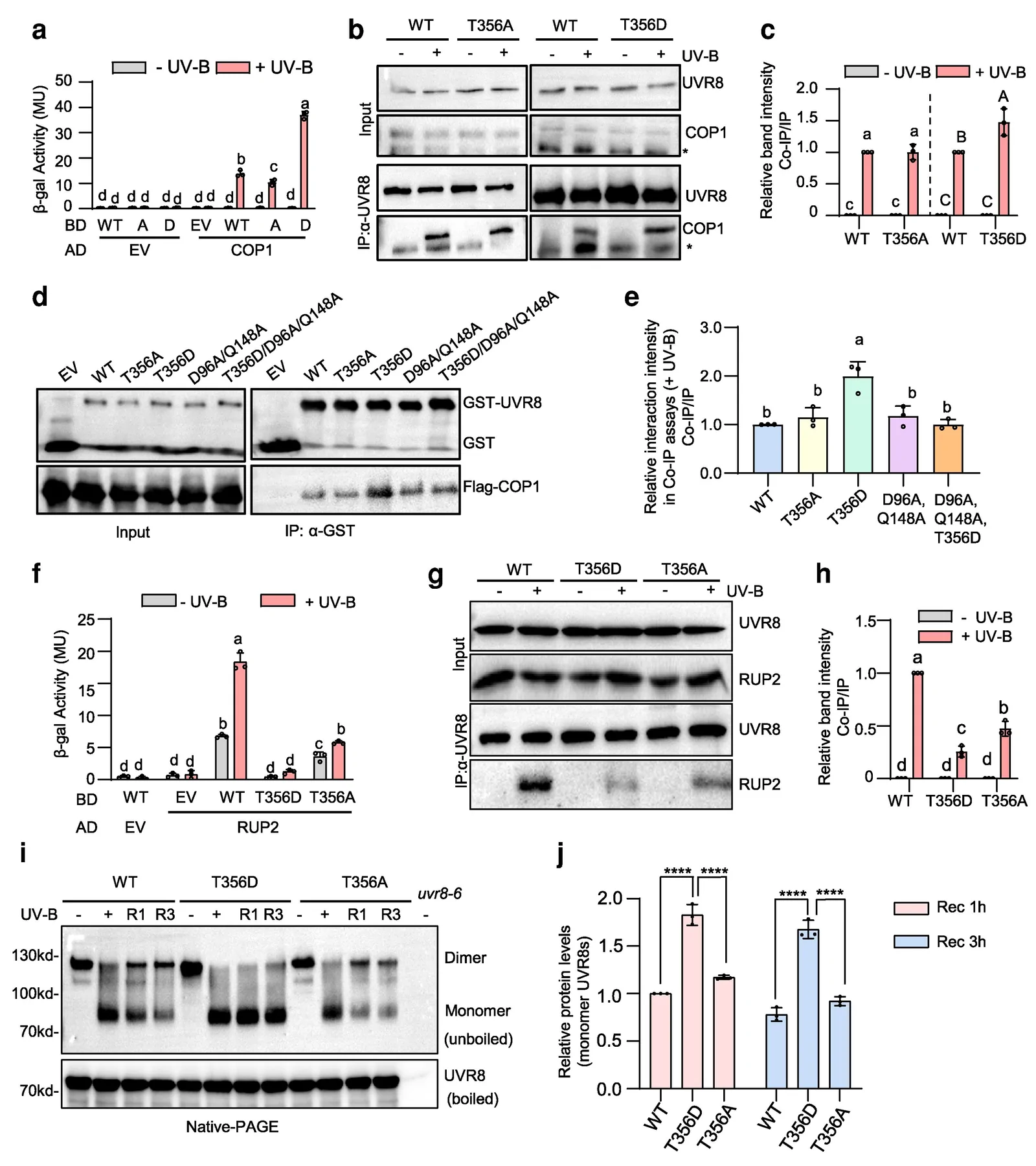
Phosphorylation of T356 affects UVR8 binding to its key protein partners like COP1 and RUP2. (a) UVR8^T356D^ interacts stronger with COP1 than wild-type UVR8 in yeasts. Quantitative yeast 2-hybrid assays were performed. Means and standard errors from 3 biological replicates are shown. β-gal, β-galactosidase; MU, Miller units. Empty vector (EV), wild-type UVR8 (WT), UVR8^T356A^ (a), UVR8^T356D^ (d). (b, c) UVR8^T356D^ interacts stronger with COP1 than wild-type UVR8 in planta. (b) Co-immunoprecipitation of COP1 with UVR8 (using the anti-UVR8^426-440^ antibody) from 7-d-old WT (*35S:YFP-UVR8/uvr8-6*), T356A (*35S: YFP-UVR8^T356A^/uvr8-6*), and T356D (*35S:YFP-UVR8^T356D^/uvr8-6*) seedlings. Seedlings were irradiated with broadband UV-B for 30 min (+) or not (−). The asterisk indicates a cross-reacting band. (c) Relative co-immunoprecipitated COP1 band intensity analysis. The co-immunoprecipitated COP1 band intensity divided by immunoprecipitated UVR8 band intensity in *35S: YFP-UVR8/uvr8-6* under UV-B was set to 1. Mean and SD from 3 independent experiments are shown. (d, e) UVR8^T356D^ has stronger interaction with COP1 than wild-type UVR8 in vitro. Binding of UVR8^T356D^ to COP1 was reduced by substitutions of UVR8 D96 and Q148 with Alanine (D96A/Q148A). (d) Pull-down of FLAG-COP1 with GST-UVR8 (using anti-GST agaroses) proteins irradiated with broadband UV-B for 15 min. EV, empty vector. (e) Relative co-immunoprecipitated FLAG-COP1 band intensity analysis. The co-immunoprecipitated FLAG-COP1 band intensity divided by immunoprecipitated GST-UVR8 band intensity was set to 1. Mean and SD from 3 independent experiments are shown. (f) UVR8^T356D^ exhibits strongly reduced interaction with RUP2 than wild-type UVR8 in yeasts. Quantitative yeast 2-hybrid assays were performed similar as that in Fig. 4a. Means and standard errors from 3 biological replicates are shown. β-gal, β-galactosidase; MU, Miller units; EV, empty vector. (g, h) UVR8^T356D^ exhibits significantly reduced interaction with RUP2 than wild-type UVR8 and UVR8^T356A^ in planta. (g) Co-immunoprecipitation of RUP2 with UVR8 (using the anti-UVR8^426-440^ antibody) from 7-d-old WT (*35S:YFP-UVR8/uvr8-6*), T356A (*35S:YFP-UVR8^T356A^/uvr8-6*) and T356D (*35S:YFP-UVR8^T356D^/uvr8-6*) seedlings. Seedlings were irradiated with broadband UV-B for 30 min (+) or not (−). The asterisk indicates a cross-reacting band. (h) Relative co-immunoprecipitated RUP2 band intensity analysis. The co-immunoprecipitated RUP2 band intensity divided by immunoprecipitated UVR8 band intensity in *35S: YFP-UVR8/uvr8-6* under UV-B was set to 1. Mean and SD from 3 independent experiments are shown. (i, j) T356D (*35S:YFP-UVR8^T356D^/uvr8-6*) exhibits slower redimerization than WT UVR8 (*35S:YFP-UVR8/uvr8-6*) and T356A (*35S:YFP-UVR8^T356A^/uvr8-6*). (i) Seedlings grown in white light for 4 d (−), then were irradiated with broadband UV-B for 30 min (+) before recovery in white light for the indicated time. R1 and R3 indicate recovery in white light for 1 and 3 h, respectively. Immunoblot analyses were performed with the anti-UVR8^410-424^ antibody. (j) Relative UVR8 monomer levels were quantified against that of WT UVR8 recovery for 1 h. Mean and SD from 3 independent experiments are shown. Statistical analyses in (a), (c), and (h) were performed with 2-way ANOVA, in (e) was performed with 1-way ANOVA with different lowercase letters indicating significant differences (*P* < 0.05), and in (j) was performed by Student’s *t*-test (****P < 0.0001).

The WD40 domain of COP1 binds to UVR8, and the co-structure of COP1^WD40^-UVR8 has been resolved (Wang et al. 2022). To explore the structural basis for the enhanced COP1 interaction, we modeled COP1^WD40^-UVR8^T356D^, COP1^WD40^-UVR8^T356A,^ and COP1^WD40^-UVR8 complexes using AlphaFold3.0. Predicted complexes of UVR8^T356D^, UVR8^T356A^, and wild-type UVR8 showed overall similarity to the solved COP1^WD40^-UVR8 structure (PDB: 7VGG), but UVR8^T356D^ introduced new hydrogen bonds between UVR8^D96^ and COP1^H369^, and UVR8^Q148^ and COP1^S418^ (Figure S14a–e). To test the direct interaction between UVR8 and COP1, we purified FLAG-COP1 with GST-UVR8 variant proteins and then performed pull-down assays to assess their physical interaction. It should be noted that broad-band UV-B was applied to ensure complete monomerization of both GST-UVR8 and GST-UVR8^T356D^ (Figure S15). GST-UVR8^T356D^ exhibited stronger binding to FLAG-COP1 than GST-UVR8 under UV-B (Fig. 4d, e). Moreover, pull-down assays with D96A and Q148A substitutions confirmed that these residues contribute strongly to the enhanced COP1 binding in UVR8^T356D^ (Fig. 4d, e). Thus, phosphorylation at T356 promotes COP1 binding, likely via introducing additional hydrogen bonds. It has been shown that UVR8 competes with HY5 for binding to COP1 (Lau et al. 2019). We, therefore, employed a yeast three-hybrid system to assess whether the UVR8 variants T356D and T356A affect COP1-HY5 association. Our results showed that, under white light conditions, expression of UVR8 or its variants had no detectable effect on COP1-HY5 interaction. However, upon UV-B exposure, UVR8 and its variants significantly suppressed COP1-HY5 interaction, with T356D exhibiting the strongest inhibitory effect (Figure S16). These findings are consistent with the enhanced COP1 binding observed for T356D.

### Phosphorylation of T356 impairs UVR8-RUP2 interaction and redimerization

RUP1 and RUP2 are key negative regulators of UVR8, and they bind to and facilitate UVR8 redimerization. Compared to RUP1, RUP2 plays a more prominent role in this process. Therefore, we tested whether T356 phosphorylation affects RUP2 binding and redimerization. Yeast two-hybrid, split-luciferase, and Co-IP assays all showed that UVR8^T356D^ essentially failed to bind RUP2 (Fig. 4f–h, Figure S17). Interestingly, UVR8^T356A^ exhibited moderately reduced RUP2 binding in comparison to wild-type UVR8 (Fig. 4f–h). Consistent with the strongly impaired binding to RUP2, the redimerization of UVR8^T356D^ was more strongly delayed than in wild-type UVR8 and UVR8^T356A^ (Fig. 4i, j). These results indicate that phosphorylation at T356 disrupts RUP2 binding. We noted that T356 is not a direct contact point for UVR8-RUP2 interaction, yet T356D essentially fails to bind RUP2. We hypothesize that phosphorylation at T356 introduces a negative charge that perturbs the local conformation, which may propagate to the RUP2-binding interface and impair its recognition. Together, phosphorylation at T356 not only facilitates UVR8 monomerization, but also delays UVR8 redimerization, shifting dimer/monomer photo-equilibrium towards monomer.

### Phosphorylation of T356 promotes UVR8 nuclear accumulation

Nuclear localization is a prerequisite for UVR8 function in UV-B signaling (Kaiserli and Jenkins 2007; Yin et al. 2016; Qian et al. 2020). Since COP1 promotes UVR8 nuclear accumulation, we examined whether T356 phosphorylation alters this process. Immunoblot analyses of nuclear and cytosolic fractions showed that UV-B triggered nuclear accumulation of YFP-UVR8 and YFP-T356A, which reverted after white-light recovery (Fig. 5a, b). In contrast, YFP-T356D accumulated more strongly in the nucleus under UV-B and was retained at higher nuclear levels during recovery in white light (Fig. 5a, b). Confocal imaging confirmed that UVR8^T356D^ seedlings displayed elevated nuclear fluorescence under UV-B and maintained higher signals during recovery compared to YFP-UVR8 or YFP-T356A (Fig. 5c, d). It appears that UV-B induced a moderate increase in nuclear YFP-UVR8 levels as detected by immunoblotting, but a clear increase in nuclear YFP fluorescence signal (Fig. 5a–c). This is likely due to the very concentrated fluorescence signal in the small nuclei. Together, these results demonstrate that T356 phosphorylation promotes UV-B-induced UVR8 dimer dissociation, enhances COP1 binding and suppresses RUP2 binding, thereby enhancing UV-B-induced UVR8 nuclear accumulation and signaling output (Fig. 5e).

**Figure 5:**
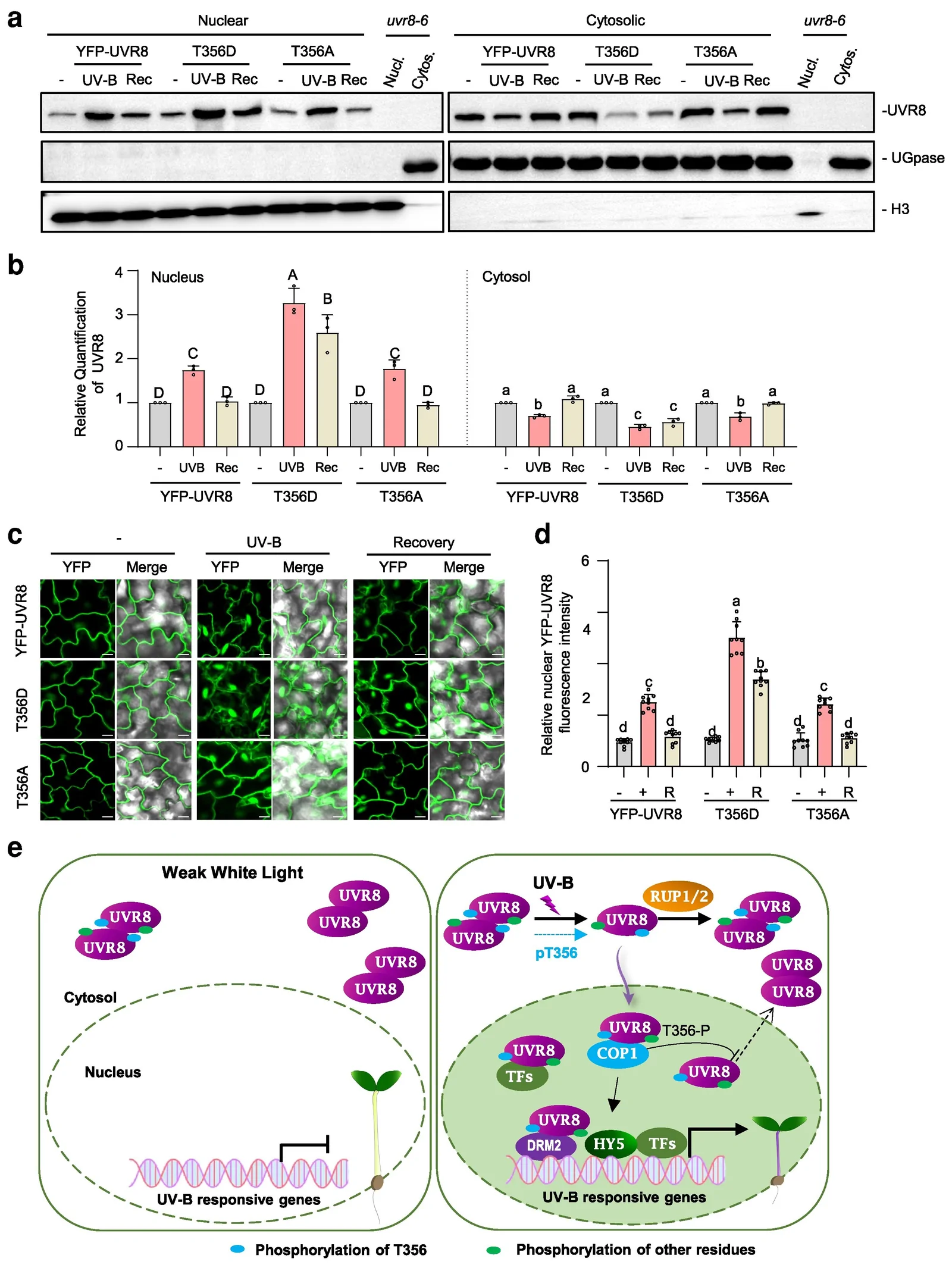
Phosphorylation of T356 enhances UV-B-induced UVR8 nuclear accumulation. (a, b) T356D seedlings exhibit stronger UV-B-induced UVR8 nuclear accumulation and nuclear retention. (a) Immunoblot analyses of UVR8, histone H3 (nuclear control), and UGPase (cytosolic control) in nuclear and cytosolic proteins of 7-d-old YFP-UVR8 (*35S:YFP-UVR8/uvr8-6*), T356D (*35S:YFP-UVR8^T356D^/uvr8-6*) and T356A (*35S:YFP-UVR8^T356A^/uvr8-6*) plants grown in white light without (−) or with UV-B for 24 h (UV-B), and UV-B-irradiated plants were recovered under white light for 3 h (Rec). (b) Relative quantification of UVR8 protein levels in nucleus and cytosol was performed by comparison to that under white light (−) for each genotype. Mean and SD from 3 experiments are shown (*n* = 3). The different uppercase letters in nuclear proteins and lowercase letters in cytosolic proteins, respectively, represent significant differences as determined by 2-way ANOVA with Tukey's HSD test (*P* < 0.05). (C) YFP fluorescence in cotyledon adaxial epidermis of 4-d-old *35S:YFP-UVR8/uvr8-6* (#17), *35S:YFP-UVR8^T356D^/uvr8-6* (#5), and *35S:YFP-UVR8^T356A^/uvr8-6* (#19) seedlings grown under white light (−) or white light supplemented with UV-B for 24 h (UV-B), or recovery under white light for 3 h after UV-B treatment (Recovery), Bars = 10 μm. (d) Relative nuclear fluorescence intensities were normalized against that under white light for each genotype. Mean and SD are shown (*n* = 9). The different lowercase letters represent significant differences in nuclear proteins, as determined by 2-way ANOVA with Tukey's HSD test (*P* < 0.05). (e) The proposed working model for T356 phosphorylation on UV-B sensing and signaling. Under white light, UVR8 proteins exist predominantly as inactive homodimers in cytosol, and a small portion of UVR8 is phosphorylated at several residues including T356. UV-B enhances UVR8 phosphorylation at multiple residues including T356. UV-B serves as the primary trigger for UVR8 dimer dissociation. RUP1/2 interact with the UVR8 monomer and accelerate UVR8 redimerization. T356 phosphorylation functions as a modulatory node that facilitates UVR8 monomerization, inhibits UVR8-RUP2 binding, and delays UVR8 redimerization, shifting dimer/monomer equilibrium towards monomer. T356 phosphorylation is depicted as a modulatory node that fine-tunes dimer/monomer equilibrium and protein interactions, rather than an essential on/off switch. Monomeric and phosphorylated UVR8 (including pT356) translocate into nucleus, where it binds strongly to COP1, leading to enhanced UVR8 nuclear accumulation and nuclear retention. UVR8-COP1 interaction also inactivates COP1, leading to the stabilization of HY5 that orchestrates the transcription of downstream UV-B response genes. In the nucleus, UVR8 also binds to other proteins including several transcription factors (TFs) and a DNA methyltransferase DRM2 to regulate gene transcription and UV-B signaling. Whereas nonphosphorylated UVR8 dominates in cytosol, nuclear UVR8 is exclusively phosphorylated, suggesting that phosphorylated monomer represents the active state of UVR8 in UV-B signaling. Small blue and green circles indicate phosphorylation at T356 and other residues of UVR8, respectively.

## Discussion

Our study identifies phosphorylation of UVR8 at T356 as a previously unrecognized regulatory mechanism that regulates the UVR8-mediated UV-B signaling. While phosphorylation has long been known to modulate the activities of major plant photoreceptors—including phytochromes and cryptochromes (Wong et al. 1986; Shalitin et al. 2002; Medzihradszky et al. 2013; Liu et al. 2017, 2023; Zhou et al. 2018; Zhao et al. 2023)—its functional relevance in UVR8 core signaling has remained unresolved. We show that T356 phosphorylation critically regulates the UVR8 dimer/monomer status by facilitating the UV-B-induced dimer dissociation and suppressing monomer-to-dimer reversion, thereby shifting the photo-equilibrium toward the monomeric, signaling-competent state (Figs. 3f, 4i, j). This shift likely enables plants to boost UV-B responsiveness under low-fluence conditions such as canopy sunflecks (Findlay and Jenkins 2016; Moriconi et al. 2018) and laboratory conditions (Figure S18). Given that UV-B-induced UVR8-COP1 interaction drives UVR8 nuclear retention (Favory et al. 2009; Cloix et al. 2012; Yin et al. 2016; Lau et al. 2019; Fang et al. 2022), T356 phosphorylation further enhances UVR8 signaling output by strengthening UVR8-COP1 binding and promoting nuclear localization. Correspondingly, phosphomimetic variants UVR8^T356D^ in Arabidopsis and SlUVR8^T360D^ in tomato exhibit consistently heightened activity across all UV-B responses analyzed. Together, these findings demonstrate that T356 phosphorylation integrates control of UVR8 dimer/monomer status, protein-protein interactions, and subcellular trafficking, thereby establishing a new regulatory layer in plant UV-B signal transduction.

The transition of UVR8 from a homodimer to an active monomer is a critical step in UV-B perception (Rizzini et al. 2011; Christie et al. 2012; Wu et al. a 2012). Two distinct phosphomimetic substitutions at T356, either Aspartate (T356D) or Glutamate (T356E) (Fig. 3a, b), accelerate UV-B-induced UVR8 monomerization, indicating that phosphorylation at T356 is important to UVR8 conformation. In plants, monomeric UVR8 can reassociate to form homodimers, a process facilitated by RUP proteins (Heijde and Ulm 2013; Zhang et al. 2021). Phosphorylation of T356 prevents RUP2 binding, thereby inhibiting redimerization in vivo. Conversely, dephosphorylation at T356 does not affect UVR8 dimer dissociation but slightly delays redimerization, consistent with modestly reduced RUP2 interaction. Notably, the UV-B signaling activity of the UVR8^T356A^ lines was not affected by the slightly delayed redimerization of UVR8^T356A^ (Figs. 2 and 4i, j), suggesting that only strongly altered binding to RUP proteins can lead to altered UV-B signaling. Consistent with this notion, Arabidopsis *rup1* single mutants are similar to WT in UV-B signaling, and only *rup1 rup2* double mutants exhibit strongly enhanced UV-B signaling (Gruber et al. 2010). The phospho-loss mutant UVR8^T356A^ mediates normal UV-B signaling, which is attributed to its normal monomerization, COP1 binding, and nuclear localization. These observations suggest that, besides T356, other phosphorylation residues may contribute to fine-tuning UVR8 dimer/monomer status. Supporting this notion, multiple putative phosphorylation residues have been identified previously (Liu et al. 2024) and in this study. Although UVR8^T356A^ did not exhibit severe defects, this does not diminish the physiological relevance of T356 phosphorylation. Rather, we reason that under the relatively strong UV-B conditions commonly used in laboratory settings, the UVR8 signaling pathway possesses intrinsic robustness, and T356 phosphorylation serves primarily to fine-tune the sensitivity threshold of the response. The subtle phenotype of T356A underscores the functional redundancy among multiple phosphorylation sites and the overall robustness of the core UV-B signaling cascade. Regarding the subtle phenotype of T356A, another possible explanation is that a threshold level of UVR8 phosphorylation must be reached within a population of molecules for a biological effect to become apparent. This threshold may be achieved more consistently in the T356D mutant, which could account for the stronger phenotype observed. Conversely, the hyper-sensitive phenotype of T356D reveals the physiological significance of this modification under suboptimal UV-B conditions, where it lowers the threshold for monomerization, enabling plants to mount a rapid response even under weak UV-B signals. In addition to dimer/monomer status, COP1-binding plays a critical role in UVR8-mediated UV-B signaling. T356A retains sufficient COP1 binding and nuclear localization to support signaling, whereas T356D enhances these processes. Supporting this notion, under natural light conditions, such as sunflecks or diurnal photoperiods, UVR8 exists in a dimer/monomer photo-equilibrium (Findlay and Jenkins 2016; Moriconi et al. 2018). Our results indicate that T356 phosphorylation shifts this equilibrium toward the monomeric, signaling-competent form, thereby enhancing UV-B responsiveness.

UVR8 is highly abundant in plant cells, with most proteins residing in the cytosol and only a small fraction translocating into the nucleus under UV-B (Kaiserli and Jenkins 2007; Qian et al. 2016; Yin et al. 2016). Consistent with previous observations (Liu et al. 2024), we found that only a minor fraction of UVR8 is phosphorylated, and UV-B enhances this modification. Importantly, nuclear UVR8 is phosphorylated (Fig. 1c), suggesting that even at low abundance, the phosphorylated nuclear pool is sufficient to drive UV-B-mediated photomorphogenesis and stress acclimation (Kaiserli and Jenkins 2007; Yin et al. 2016; Qian et al. 2020). In contrast, cytosolic UVR8 shows a lower phosphorylation level compared to the nuclear pool and is less responsive to UV-B treatment. The precise nucleo-cytoplasmic distribution of UVR8^T356-P^ in wild-type plants remains unclear due to the lack of a phospho-specific antibody. However, complementation analyses indicate that the phosphomimetic UVR8^T356D^ exhibits hypersensitivity to UV-B, supporting the notion that UVR8^T356-P^ is at least partly nuclear. Consistently, mass spectrometry with nuclear protein extracts indicates that UV-B enhances T356 phosphorylation in the nucleus.

In the nucleus, UVR8 interacts with multiple partners, including COP1, RUP1/2, several transcription factors, and the DNA methyltransferase DRM2, to regulate gene expression and UV-B responses (Favory et al. 2009; Gruber et al. 2010; Yang et al. 2018; Liang et al. 2019; Qian et al. 2020; Jiang et al. 2021; Li et al. 2024). Because nuclear UVR8 is phosphorylated, these interactions are likely influenced by UVR8 phosphorylation. Indeed, we show that phosphorylation at UVR8 T356 enhances COP1 binding. Recently, UVR8 was also reported to interact with LOX1 in the cytoplasm to regulate stomatal movement (Liu et al. 2025), raising the question of whether UVR8 phosphorylation affects cytosolic interactions. Structural analyses indicate that T356 phosphorylation strengthens COP1 binding by introducing new hydrogen bonds, including UVR8^D96^-COP1^H369^ and UVR8^Q148^-COP1^S418^ (Fig. 4d, Figure S14). Notably, previous studies showed that mutations at D96 and D107 (D96N, D107N) produce constitutive photomorphogenic phenotypes and enhance COP1 interaction (Podolec et al. 2021b), highlighting the critical role of D96 in mediating binding. Whether additional residues contribute to the enhanced UVR8^T356D^-COP1 interaction remains to be determined.

Mass spectrometry analysis in this study identified 10 phosphorylation sites on UVR8, complementing previous reports of additional potential residues (Liu et al. 2024), highlighting the complexity and diversity of UVR8 phosphorylation. The phosphorylated UVR8 residues identified in our study only partially overlap with those reported by Liu et al. (2024), with 4 common residues (S105, S157, S160, and S414) (Figure S3). In addition, we identified several novel phosphorylation sites, most notably T356. We attribute these discrepancies primarily to 2 experimental differences: (i) seedling age—Liu *et al*. used 10-day-old seedlings, whereas we used 5-day-old seedlings, and developmental stage may influence the phosphorylation status of UVR8; and (ii) enrichment strategy—Liu *et al*. employed GFP-tag-based affinity purification, while we used a native UVR8-specific antibody. Among these, phosphorylation of S402, located at the C-terminus, was shown to modulate late UV-B responses, such as the promotion of flavonoid biosynthesis, without affecting early signaling events including monomerization, redimerization, or HY5 transcription (Liu et al. 2024). In contrast, phosphorylation at T356 exerts broad and profound effects on UVR8, impacting monomerization, redimerization, key protein interactions, nuclear localization, *HY5* transcription, hypocotyl inhibition, flavonoid accumulation, and UV-B stress acclimation and tolerance. Notably, COP1 binding, a critical early step in UVR8-mediated core signaling, is enhanced by T356 phosphorylation but not by S402, illustrating that phosphorylation at distinct residues can selectively regulate different aspects of UVR8 function. Mass spectrometry also identified Y407 and T414 as putative phosphorylation sites, all positioned near the Val-Pro motif critical for COP1 binding (Cloix et al. 2012; Yin et al. 2015; Lau et al. 2019). Whether phosphorylation at these residues modulates UVR8 signaling remains an intriguing question for future studies. Whether the T356D/A mutations affect UVR8 phosphorylation status remains an open question. Phos-tag SDS-PAGE is not well-suited for addressing this due to the low abundance of the phosphorylated subpopulation and the presence of multiple phosphorylation sites; targeted mass spectrometry-based phosphopeptide quantification would be a more appropriate approach for future studies.

UVR8 exists in both dimeric and monomeric forms in plants, raising the question of which form is phosphorylated and how this modification relates to activity. Detection of phosphorylated UVR8 in wild-type plants under white light, as well as in the constitutive dimer UVR8^W285F^ mutant, provides strong evidence that UVR8 can be phosphorylated in its dimeric state. Consistently, T356 is located at the dimer interface surface and is accessible to kinases (Fig. 3h). Analysis of the sequence flanking T356 (RGTNGQLGI) revealed a conserved R/K-X-T/S motif, a typical recognition site for AGC family kinases. Whether these kinases or others, including PPKs, which are known to be involved in light signaling, phosphorylate UVR8 awaits further investigation. Upon UV-B exposure, monomeric UVR8 accumulates in the nucleus (Fang et al. 2022), where only the phosphorylated form is detected, while non-phosphorylated UVR8 remains cytosolic (Fig. 1c). These observations indicate that both phosphorylated dimers and monomers coexist in plant cells. Given that nuclear monomers mediate UV-B responses (Kaiserli and Jenkins 2007; Yin et al. 2016; Qian et al. 2020), we propose that the phosphorylated monomer represents the active signaling state of UVR8.

In conclusion, our study establishes that UVR8 phosphorylation is dynamically regulated by UV-B and primarily accumulates in the nucleus. UV-B-enhanced phosphorylation at T356 of UVR8 emerges as a critical mechanism that promotes monomerization, inhibits redimerization, shifts the dimer/monomer photo-equilibrium toward the monomer, enhances COP1 binding, and promotes nuclear localization, collectively amplifying UV-B signaling and stress tolerance (Fig. 5e). These findings reveal a previously unappreciated layer of post-translational control over UVR8 and provide mechanistic insights into how plants fine-tune UV-B perception and photoreceptor activity.

## Materials and methods

### Plant materials and treatments

*Arabidopsis thaliana uvr8-6* and *uvr8-7* mutant alleles are in the Columbia (Col) and Wassilewskija (Ws) background, respectively (Favory et al. 2009). The *35S:UVR8/uvr8-7*, *35S:UVR8^W285A^/uvr8-7*, and *35S:UVR8^W285F^/uvr8-7* transgenic lines were described before (Heijde et al. 2013). Tomato (*Solanum lycopersicum*) mutant allele *Sluvr8 #1* used in this study was previously generated in the *Ailsa Craig* background (Liu et al. 2020).

To generate Arabidopsis transgenic lines expressing UVR8 variants, the wild-type *UVR8*, *UVR8^T356A^*, *UVR8^T356D^*, *UVR8^T356E^*, *UVR8^S105A^*, *and UVR8^S105D^* coding sequences were introduced into pDONR221 (Thermo Fisher Scientific) via site-directed mutagenesis. Primers used were listed in Table S1. Sequences of *UVR8*, *UVR8^T356A^*, *UVR8^T356D^*, *UVR8^S105A^*, *and UVR8^S105D^* were inserted into the Gateway-compatible binary vector pB7WGY2, and sequences of wild-type *UVR8*, *UVR8^T356A,^* and *UVR8^T356E^* were inserted into vector pB2GW7 via Gateway cloning (Karimi et al. 2002). To generate tomato transgenic lines *35S: SlUVR8-GFP/Sluvr8* and *35S:SlUVR8^T360D^-GFP/Sluvr8*, the *SlUVR8* and *SlUVR8^T360D^* coding sequences were cloned into the pMV2 vector. The constructs were transformed into the *Sluvr8* plant cotyledons using Agrobacterium tumefaciens as described before(Liu et al. 2020).

Soil-grown Arabidopsis seedlings were cultured in long-day (16 h/8 h) growth chambers at 23 °C/20 °C (day/night) under a white light intensity of 100 μmol m^−2^ s^−1.^ Tomato plants were grown in the same growth chamber with the same photoperiod under a white light intensity of 150 μmol m^−2^ s^−1^. Arabidopsis seeds were surface-sterilized using ethanol and Triton X-100 and then sown on half-strength Murashige and Skoog (1/2 MS, Duchefa Biochemie) basal salt medium containing 1% (w/v) sucrose and 1% (w/v) agar, and seeds were stratified for 2 d in the dark at 4 °C.

For measurement of hypocotyl length and anthocyanin contents, seedlings were grown under white light (3.6 μmol m^−2^ s^−1^, measured by LI-250A Light Meter) or the same white light supplemented with narrowband UV-B (Philips TL20W/01RS; 1.5 μmol m^−2^ s^−1^). For the dimer/monomer experiments, seedlings were grown under white light (3.6 μmol m^−2^ s^−1^) and then treated with broadband UV-B similarly as described previously (Philips TL40W/12RS) (Liu et al. 2020). For RT-qPCR experiments, plants were grown under white light (3.6 μmol m^−2^ s^−1^) and then treated with narrowband UV-B (1.5 μmol m^−2^ s^−1^) for the indicated time.

### Isolation of nuclear and cytosolic proteins

Nuclear and cytosolic proteins from 4-day-old Arabidopsis seedlings were isolated similarly as described previously (Yin et al. 2016). Total proteins were extracted using a lysis buffer containing 20 mM Tris-HCl (pH 7.4), 25% glycerol, 20 mM KCl, 2 mM EDTA, 2.5 mM MgCl_2_, 250 mM sucrose, 1 mM DTT, and 1 mM PMSF. The homogenate was sequentially filtered through 3 layers of 40 μm nylon mesh. The filtered homogenate was centrifuged at 1,500 *×g* for 10 min at 4 °C to pellet the nuclei. The supernatant (cytosolic fraction) was collected, and the nuclear pellet was washed 3 times with 20 mL of nuclei wash buffer (20 mM Tris-HCl, pH 7.4, 25% glycerol, 2.5 mM MgCl_2_, and 0.2% Triton X-100). Subsequently, the nuclei were washed with 10 mL nuclei resuspension buffer (20 mM Tris-HCl, pH 7.4, 25% glycerol, 2.5 mM MgCl_2_), then boiled in 5-fold Laemmli buffer, and finally dissolved in the lysis buffer.

### UVR8 Dimer/monomer analysis with native PAGE and SDS-PAGE

To detect UVR8 by native PAGE gel immunoblot analysis, the proteins were extracted using an extraction buffer consisting of 50 mM Tris-HCl (PH = 7.4), 150 mM NaCl, 5 mM EDTA, 0.1% Tween 20, 10 mM DTT, and 1% [v/v] Protein Inhibitor Cocktail (Sigma). Loading buffer (250 mM Tris-HCl, 25% [v/v] glycerol, 0.5% [w/v] bromophenol blue) was added to the non-boiled extracts before loading onto blue native gels (E302-01, Vazyme). These Native PAGE gels are cast and run without SDS. This approach preserves the native conformation of the protein complex, allowing us to assess the steady-state oligomeric status of UVR8 in its near-physiological state under different light conditions. After electrophoretic separation in blue native gels in non-SDS running buffer (25 mM Tris, 190 mM glycine), proteins were transferred to PVDF membranes according to the manufacturer's instructions (Bio-Rad, Semi-Dry and Rapid Blotting Systems).

To detect UVR8 by 8% [w/v] SDS-PAGE gel immunoblot analysis, plant total proteins were extracted using an extraction buffer containing 50 mM Tris-HCl (pH = 7.4), 150 mM NaCl, 5 mM EDTA, 0.1% [v/v] Tween 20, and 1% [v/v] Protein Inhibitor Cocktail (Sigma). The protein extracts were boiled at 100 °C for 5 min or not and then loaded onto SDS-PAGE gels. After electrophoresis, proteins were transferred to PVDF membranes according to the manufacturer's instructions (Bio-Rad).

For immunoblot analyses, anti-UVR8^426–440^ (Favory et al. 2009), anti-UVR8^410–424^(Kaiserli and Jenkins 2007), anti-SlUVR8^415–429^ (Liu et al. 2020), anti-CHS (PAB09104; Orizymes Biotechnologies), anti-COP1 (A18844; Abclonal), anti-RUP2 (Ren et al. 2019), anti-H3 (ab1791; Abcam), anti-Ugpase (AS05086; Agrisera), and anti-GAPDH (60004-1-PBS; Proteintech), anti-GFP (66002-1-Ig; Proteintech) were used as primary antibody. Anti-Rabbit (Abmart) and Anti-mouse (Abmart) secondary antibodies were used as required. Protein signals were detected with the LumiBest ECL substrate solution kit (Share-bio).

### *In vivo* phosphorylation analysis

*In vivo* phosphorylation assays using phos-tag SDS-PAGE gel (NARD Institute) were performed as described previously (Wang et al. 2021). Total protein extracts were separated on an 8% [w/v] phos-tag SDS-PAGE gel with 50 μM Phos-tag^TM^ Acrylamide and 10 μM MnCl_2_. After electrophoresis, the gel was washed 3 times with a transfer buffer containing 10 mM EDTA for 10 min each, followed by a final wash with transfer buffer for another 10 min before transferring to a PVDF membrane. Lambda-phosphatase treatments (New England Biolabs) were applied by adding the enzyme and its reaction buffer to the total proteins according to the manufacturer's instructions.

### Co-immunoprecipitation analysis

Total proteins were extracted using the extraction buffer (50 mM Tris-HCl, pH 7.4, 150 mM NaCl, 2 mM EDTA, 2 mM Na3VO4, 2 mM NaF, 20 mM β-glycerophosphate, 10% glycerol, 1 mM phenylmethylsulfonyl fluoride, 0.1% Tween 20, and 1×Protease Cocktail). Extracts were incubated with an anti-UVR8^426–440^ antibody for 2 h, followed by incubation with protein A beads for 1 h. Beads were washed 3 times with the extraction buffer. The immunoprecipitated proteins were analyzed by SDS-PAGE gel and detected using anti-UVR8^426–440^, anti-COP1, and anti-RUP2 antibodies.

### Extraction and analysis of anthocyanins

Plants were grown under the previously described conditions for 4 d. Approximately 300 mg of fresh seedlings were ground, and pigments were extracted in 1 mL of methanol with 1% [v/v] HCl at 4 °C for at least 1 h. Clear supernatants were collected, and absorbance was measured at 530 and 657 nm. The content of anthocyanins was calculated as (A530-0.25*A657)/m, where m indicates seedling fresh weight.

### Sample preparation for mass spectrometry (MS) assays

4-day-old Col-0, *uvr8-6* plants were collected and ground into powder, and total proteins were extracted using a buffer containing 50 mM Tris-HCl, pH 7.4, 150 mM NaCl, 2 mM Na3VO4, 2 mM NaF, 20 mM β-glycerophosphate, 10% glycerol, 0.1% Tween 20, and 1×Phostase cocktail, and co-immunoprecipitated by anti-UVR8^426–440^ antibody. The proteins were separated by SDS-PAGE, and the gel was stained using the Pierce™ Silver Stain Kit (Thermo Fisher) according to the manufacturer's protocol. UVR8 protein bands were identified by comparing the band patterns of wild-type and *uvr8 mutant*. The identified UVR8 bands were excised and subjected to mass spectrometry to identify phosphorylation residues.

The gel slices were de-stained with freshly prepared de-staining buffer in the dark until the gel lost its silver stain color. The de-staining buffer was replaced with 100% acetonitrile, and the gel slices were agitated at room temperature for 10–15 min. Then DTT solution was added and incubated for 20 min, and then further incubated in a freshly prepared iodoacetamide solution at room temperature in the dark for 15 min. Trypsin solution (Promega, typically 0.5–2 ng/μl) was used to digest at 37 °C overnight. Next, samples were sonicated for 5 min in the extraction buffer and then incubated at 37 °C for 30 min. Extraction solution was collected before the next 5 min of sonication in 100% acetonitrile and incubation at 37 °C for 30 min. This solution was mixed with the previous extraction solution, and the combined extract was dried using a centrifugal concentrator (keeping the sample temperature below 37 °C). The sample was dried and dissolved in 10 μl of 0.1% formic acid in water with agitation before mass spectrometry analysis.

### LC-MS/MS analysis of phosphorylation peptides and data processing

Phosphorylated peptides were analyzed using online nanoflow liquid chromatography-tandem mass spectrometry (LC-MS/MS) on a Q Exactive Plus quadrupole orbitrap mass spectrometer (Easy nLC1200/Q Exactive Plus, Thermo Fisher Scientific). A 1 μl sample volume was introduced by an autosampler onto a 2 cm pre-column (75 μm ID × 360 μm OD) packed with C18 reversed-phase silica (5 μm particles, 200 Å pore size, DIKMA-inspireTM) using 0.1% formic acid in water (buffer A) at a flow rate of 1 μl/min for 5 min. The peptides were then separated on a 15 cm analytical column (75 μm ID × 360 μm OD) packed with C18 silica (3 μm particles, 100 Å pore size, DIKMA-inspireTM) at a consistent flow rate of 300 nl/min. A gradient separation was performed as follows: buffer B (0.1% formic acid in acetonitrile) increased from 3% to 6% over the first 5 min, then gradually increased to 22% over the next 36 min. It was further increased to 35% over the subsequent 9 min, followed by a rapid rise to 95% in 3 min, and maintained at 95% for 7 min. The total program run was 60 min. Full scan mass spectra were collected in the m/z range of 350–1,800 using the Orbitrap at a resolution of 70,000 (at m/z 200), following the accumulation of ions to a target value of 3 × 10^6^, based on predictive automatic gain control (AGC) from the preceding full scan. Dynamic exclusion was set for 60 s.

Data from the samples were analyzed using the Proteome Discoverer software (version 3.0). The search parameters were set as follows: the precursor mass tolerance was 10 ppm, and the fragment mass tolerance was 0.020 u. The fixed modification included phosphorylation (P). The digestion was performed using trypsin, allowing for up to 2 missed cleavage sites. T356 phosphopeptide signal intensity is calculated as the (Phosphopeptide ion intensity)/(Total protein ion intensity). A false discovery rate (FDR) threshold of ≤1% was applied for both protein and peptide identifications.

### Real-time quantitative PCR analysis

RNA was extracted using the Plant RNA Kit (Vazyme), cDNA synthesis was performed with the EasyScript® One-Step gDNA Removal and cDNA Synthesis SuperMix (Vazyme), and RT-qPCR was conducted using Hieff® qPCR SYBR® Green Master Mix (Vazyme) on the CFX96 Real-Time PCR Detection System (Bio-Rad) following the manufacturer's protocol. RT-qPCR was performed in 20μL reaction volumes with gene-specific primers (Table S1). For the calculation of relative transcription levels, the delta of threshold cycle (△Ct) values was calculated by subtracting the arithmetic mean Ct values of the target genes from the normalizing Actins. The relative transcript level (2^−△△Ct^) was calculated from 3 independent experiments.

### Protein purification from *E. coli*

The full-length coding sequences of wild-type *UVR8*, *UVR8^T356A^*, *UVR8^T356D^*, *UVR8^D96A, Q148A^*, and *UVR8 ^T356D, D96A, Q148A^* were cloned into pGEX-4T (GST-tagged) vectors; full-length *UVR8* and *UVR8^T356D^* sequences were cloned into pET-15d (His-tagged) vectors; then transformed into *Escherichia coli* (BL21) strain. The strains were first cultured at 37 °C to OD_600_ = 0.6, and then the proteins were expressed at 16 °C for 16 h using 0.5 mM IPTG. Cells were harvested and lysed in 1×PBS buffer with Tiechui cracking buffer (ACE Biotechnology) for 5 min, then centrifuged to collect the supernatant. Proteins were purified by incubating the supernatant with GST and His agarose, washing with 1×PBS 5 times. Finally, the proteins were eluted from the GST or His agarose with 10 mM GSH solution or 10 mM imidazole solution.

### Size-exclusion chromatography analysis

The dimer and monomer of UVR8 and UVR8^T356D^ were determined by size-exclusion chromatography analysis. Purified protein samples from *E.coli* were injected into a Superdex 200 Increase 10/30 GL column (GE Healthcare) equilibrated with buffer containing 25 mM Tris-HCl (pH 8.0), 150 mM NaCl, and 5 mM DTT. Data analysis was performed using GraphPad Prism8.

### Yeast two-hybrid and three-hybrid assays

The *UVR8*, *UVR8^T356A^*, and *UVR8^T356D^* coding sequences were cloned into pGBKT7 and transformed into the Y2H Gold strain (Clontech). The *UVR8*, *UVR8^T356A^*, *UVR8^T356D^*, *COP1,* and *RUP2* sequences were cloned into pGADT7 and transformed into the Y187 strain (Clontech). After mating, yeast diploids were selected on the SD/-Leu/-Trp medium for 2-day non-selective growth in the dark at 30 °C, followed by selection on the SD/-Leu/-Trp/-His medium for 3-day selective growth in the dark or under continuous narrowband UV-B (1.5 μmol m^−2^ s^−1^). For quantification, chlorophenol red-β-D-galactopyranoside (CPRG, Roche Applied Science) was used as the substrate to detect β-galactosidase activity as described previously (Yin et al. 2015). Primers used to construct the vectors are listed in Table S1.

For yeast three-hybrid assays, the prey protein COP1 was expressed as a fusion protein with the GAL4 activation domain in the pGADT7 vector. The vector pBridge was used to express the GAL4 DNA-binding domain fused to the bait protein HY5 and a bridge protein (UVR8 or UVR8^T356D^, or UVR8^T356A)^. The interaction between COP1 and HY5 was assayed in the presence or absence of Met (1 mM). The bait and prey vectors were cotransformed into the AH109 strain. Yeast diploids were selected on the SD/-Leu/-Trp medium for 2 d non-selective growth in the dark at 30 °C followed by selection on the SD/-Leu/-Trp/-His/+Met or SD/-Leu/-Trp/-His/-Met medium for 3-day selective growth in the dark or under continuous narrowband UV-B (1.5 μmol m^−2^ s^−1^). Primers used to construct the vectors are listed in Table S1.

### Split-luciferase complementation assay

*UVR8*, *UVR8^T356A^*, and *UVR8^T356D^* and *RUP2* sequences were cloned into pJW771/nLUC vectors, while *COP1* and *UVR8*, *UVR8^T356A^*, and *UVR8^T356D^* sequences were cloned into pJW772/cLUC vectors, followed by transformation into Agrobacterium. The combinations of the indicated insert-nLUC and insert-cLUC were transiently expressed in *N. Benthamiana* leaves. Afterwards, plants were cultured in the dark for 36 h and irradiated without or with 12 h of narrowband UV-B after infiltration. Luciferase activity was measured using a CCD camera (Tanon) upon application of firefly D-luciferin (NanoLight).

### In vitro pull-down assay

For in vitro UVR8s-COP1 pull-down, equal amounts (200 ng) of purified GST-empty vector, GST-UVR8, GST-UVR8^T356A^, GST-UVR8^T356D^, GST-UVR8^D96A, Q148A^, and GST-UVR8 ^T356D,D96A,Q148A^ proteins were incubated and irradiated with broadband UV-B for 20 min on ice, and then pre-incubated in a binding buffer (20 mM Tris-HCl, pH 7.5, 150 mM NaCl) with GST agarose (YISHENG) at 4 °C for 2 h with gentle rotation. Subsequently, 100 ng of FLAG-COP1 protein was incubated with GST-UVR8 s at 4 °C for 1 h with gentle rotation. GST agarose was spun down and washed 3 times with washing buffer (50 mM Tris-HCl, 1 mM EDTA, 0.1% Triton X-100, and 10% glycerol). Anti-COP1 and anti-UVR8 antibodies were used for immunoblotting.

### Protein structure alignment and visualization of protein interactions using PyMOL

In the study of protein structures, PDB files of the interacting proteins are retrieved from the National Center for Biotechnology Information (NCBI) website or predicted by AlphaFold 3.0 (Abramson et al. 2024). These files are then imported into PyMOL. The missing residues in the resolved COP1^WD40^-UVR8 complex (PDB:7VGG) were completed using the AlphaFold-predicted COP1^WD40^-UVR8 complex structure as a template. Once imported, the protein structures are initially presented in a default display style. To improve the visual differentiation between different proteins and their structural features, adjustments to the color and structure style are required. The display style of the proteins is changed to the “cartoon” representation. Meanwhile, the amino acids participating in hydrogen bonding are shown in the “sticks” style. The colors of the proteins and related elements can be freely adjusted. The distances between 2 interacting proteins are measured by PyMOL and labeled by hydrogen bonds. These hydrogen bonds are typically visualized as dashed lines that connect the donor and acceptor atoms.

### Subcellular localization assay and confocal fluorescence microscopy

The YFP fluorescent signals in epidermal cells of cotyledons were visualized and imaged using an Olympus Fluoview FV-3000 microscope equipped with x20/1.2 NA water-immersion objectives. YFP was excited at 488 nm and detected between 490 and 530 nm. The fluorescence intensity was measured by ImageJ software.

### Accession number

Gene sequence data from this work can be retrieved from the Arabidopsis Genome Initiative or GenBank/EMBL databases under the following accession numbers: AT5G63860 (*UVR8*), AT2G32950 (*COP1*), AT5G11260 (*HY5*), AT5G13930 (*CHS*), AT5G23730 (*RUP2*), and AT5G09810 (*ACT7*), Solyc05g018620 (*SlUVR8*), Solyc03g078400.2 (*SlActin*), Solyc08g061130.3 (*SlHY5*), Solyc09g091510.3 (*SlCHS1*).

### Statistical analysis

Column charts were drawn by the software GraphPad Prism 8. ANOVAs were conducted using SPSS software. Statistical significance was determined as *P* < 0.05 using either Student's *t*-test or one-way ANOVA, followed by Tukey's test. For experiments with more than 1 treatment, different letters represent significant differences, as determined by two-way ANOVA with Tukey's HSD test (*P* < 0.05).

## Supplementary Material

koag285_Supplementary_Data

## Data Availability

The data that support the findings of this study are available in the article and Supporting Information. Materials used in this study are available upon request to the corresponding author.
